# Evaluation of endometrial cancer epidemiology in Romania

**Published:** 2015

**Authors:** RE Bohîlțea, F Furtunescu, M Dosius, M Cîrstoiu, V Radoi, A Baroș, LC Bohîlțea

**Affiliations:** * “Carol Davila” University of Medicine and Pharmacy, Bucharest, Romania; ** National School of Public Health and Management, Bucharest, Romania; *** University Emergency Hospital Bucharest, Romania

**Keywords:** endometrial cancer, epidemiology, endometrial hyperplasia

## Abstract

Endometrial cancer represents the most frequent gynecological malignant affection in the developed countries, in which the incidence of cervical cancer has significantly decreased due to the rigorous application of screening methods and prophylaxis. According to its frequency, endometrial cancer is situated on the fourth place in the category of women’s genital-mammary malignant diseases, after breast, cervical and ovarian cancer in Romania. The incidence and mortality rates due to endometrial cancer have registered an increasing trend worldwide and also in Romania, a significant decrease of the age of appearance for the entire endometrial pathology sphere being noticed. At the national level, the maximum incidence is situated between 60 and 64 years old, the mortality rate of the women under 65 years old being high in Romania. The study evaluates endometrial cancer, from an epidemiologic point of view, at the national level compared to the international statistic data.

## Introduction

The evaluation of endometrial cancer epidemiology at the national level, compared to the statistic data reported at the international level, had as a purpose the obtaining of a broader image regarding the endometrial pathology and its repercussions with concern to the health stage of the population, the lifespan and costs of healthcare services. 

## Method

The study is descriptive and corroborates quantitative research techniques on primary and secondary data from international and national databases and also reviews of specialty literature journals.

A transversal and longitudinal descriptive analysis was undergone for the epidemiological evaluation of endometrial cancer, by using quantitative research techniques. 

For the international level, secondary data from international databases such as the following were used: WHO, International Agency for Research on Cancer, Globocan 2012: Estimated cancer incidence, mortality and prevalence worldwide in 2012 and American Cancer Institute, SEER Program, National Cancer Institute, 2013. For the national epidemiological study, a descriptive analysis was realized by taking into account the period 2009-2013 and by combining national data sources such as the following:

• Data annually published by the National Institute of Public Health – The National Center for Statistics and Informatics in Public Health, which were based on the reports of the family physicians regarding the new cases of endometrial cancer (incidence) of the patients registered in their own database, to the Public Health County Directions or to Bucharest, which were annually centralized, together with the death certificates;

• Data managed by the National School of Public Health and Management in Bucharest regarding the annual hospital admissions based on codes of diseases. Actually, these data represented the reports of the health services suppliers in the Unique Integrated Informational System (UIIS) implemented by the National Health Insurance House. Only the admissions in which the disease represented the main diagnosis on the General clinical observation sheet (Admission Form) were taken into account.

• The National Cancer Registry is a very useful source of data for research, which is currently organized on regional databases, the accessibility degree of the data varying from one region to the other. As a result, this source was only used during 2005 and 2007, beyond which, the change in the cases reporting system stopped the gathering of statistical data in this format, which was replaced with the case reports of the disease declared by the family physician and centralized by the National Health Information Center of the Ministry of Health in Romania. The database of the National Center represented the source of the statistical analysis regarding the number of new cases of endometrial cancer reported by the family physicians per year, the incidence of endometrial cancer, the annual death and mortality rates due to endometrial cancer. What is worth mentioning is the fact that the unavailability of a national register deprives the medical practice of understanding of the diseases’ evolution tendencies both at the level of the population (incidence, prevalence, survival rate) and at the individual level – the efficacy and efficiency of diagnostic and therapeutical approaches.

The incidence data were analyzed on higher age groups (15-64 years old, 65 years old and over 65 years old), this being the usual reporting modality of the family physicians. Taking into account that the studied disease is considered rare, tendencies of occurrence in absolute numbers (2009-2013) and the structure studies of the cases on the available age groups were chosen. The mortality data were available on age groups and residency areas on a five-year period. The presentation of deaths as a tendency was chosen, together with the absolute number and the structure of the deaths on four illustrative age groups, evidencing the premature mortality rates: <50 years old; 50–64 years old, 65–79 years old, 80 years old and over 80 years old. Regarding the analysis of the use of medical services, only data related to the hospital admissions were available. These data were presented on age groups, residency areas and level of education. Moreover, three risk factors mentioned in the specialty literature as being associated with endometrial cancer, respectively obesity, hypertension and type II diabetes were also followed. These risk factors were extracted in an aggregate form, according to the pathology groups and per year. The present/ absent risk factor variable was dependant on the risk factor consignment in the case report. 

## Results and Discussions

Endometrial cancer represents the most frequent malignant gynecologic disease in the developed countries, with a medium incidence of 14,7/ 100 000 women, and is situated on the 2nd place after cervical cancer, with a medium incidence of 5,5/ 100 000 women. The medium global incidence in 2012 was of 8,2/ 100 000 women, representing 319 605 cases of endometrial cancer, with a cumulative risk of 0,97%. The countries with the highest incidence rates as reported to 100 000 women are the following: Macedonia (29), Luxemburg (24,2), Czech Republic (17,97), United States of America (19,48), Guyana (22,65), Serbia (17,8), Bulgaria (17,83) and Russian Federation (16,2), the incidence being extremely low in Africa (3,5), India (2,32) and a few other countries in South America, respectively Columbia, Peru and Bolivia. The reported incidence of endometrial cancer for Romania was of 8,46/ 100 000 women in 2012 and, the medium incidence for Europe in the same year was of 13,6/ 100 000 women. 

As far as the malignant affections in women are concerned, the incidence of endometrial cancer is situated on the 6th place in the worldwide statistics, after breast, colorectal, lung, cervix and gastric cancer. Due to screening procedures and prophylaxis methods, which were rigorously applied in order to decrease the incidence of cervical cancer, endometrial cancer is situated on the 4th place in Europe, while in Romania it is situated on the 6th place, being rarely met in this category of genital diseases than cervical and ovarian cancer. According to WHO data collected from the worldwide statistics, the incidence of uterine cancer reported to 100 000 women is maximum (38,7) in the age group 70-74 years, between 65 and 69 years the incidence also being high (38,5). In Europe, the maximum incidence is of 71,8/ 100 000 women with ages between 65 and 69 years, being also very high between 70 and 74 years (71,4), the cumulative risk (0-74%) being of 1,7%. In Romania, the peak of incidence occurs in the age group of 60-64 years (42,8/ 100 000 women), the cumulated risk being of 1%. Similarly to other developed countries, in the USA, uterine cancer is the most frequent gynecological malignity with over 52 000 new cases and 8 600 deaths due to the disease in 2013. In the context in which the global incidence presents an increasing trend, the high incidence of endometrial cancer in the USA has been constant for 40 years, probably due to the efforts of decreasing the most important risk factor, obesity. According to the *US National Cancer database Surveillance, Epidemiology and End Results*, the medium incidence of uterine cancer between 2004 and 2008 was of 23,9/ 100 000 women, being higher among the Caucasians (24.8/ 100 000 women), compared to the Afro-Americans (20,9/ 100 000 women), Hispanic (18,9/ 100 000) or Asian or of the Pacific Islands (18,2/ 100 000 women). However, the mortality rate is almost twice as higher in the Afro-Americans (7,1 versus 3,9/ 100 000 women), possibly due to the high incidence of aggressive subtypes but also the reduced access to quality health services [**1**]. The medium age for the diagnosis of uterine cancer in the USA is 61 years. According to the American Cancer Society, the probability of a woman to develop endometrial cancer during lifetime is of 1 in 37, the risk being higher for breast cancer (1 in 8 women), lungs cancer (1 in 16 women) and colorectal cancer (1 in 22 women). The prevalence of endometrial cancer at the global level, calculated by WHO, in 2012, and reported to 100 000 women was of 10,9 per 1 year, 30,1 per 3 years and respectively 46,8 per 5 years. For Europe, the prevalence rate per year is of 24.7, per 3 years is of 67.7 and per 5 years is of 104.4 in 100 000 women. According to WHO statistics, the prevalence of endometrial cancer in Romania is of 14,4/ 100 000 women per year, 39,7/ 100 000 women per 3 years and respectively 61,7/ 100 000 women per 5 years. The states with the highest prevalence of endometrial cancer per 5 years in 100 000 women are the following: Luxemburg (225,8), Barbados (229,5), Macedonia (205,7), Czech Republic (153,0), United States of America (155,56), Canada (137,5), Norway (148,2), Sweden (144,46), Finland (146,4), Bulgaria (147,2) and Serbia (137,4). In the areas with a reduced incidence of the disease, the prevalence is below 10,2/ 100 000 women. The worldwide prevalence of the cases of endometrial cancer per 5 years is higher in the developed countries (52,5%) compared to the underdeveloped ones (46,5%). 

The worldwide medium mortality rate is of 1,8/ 100 000 women, with a cumulated risk of 0,21%. In 2012, 76 160 women died worldwide due to endometrial cancer, among whom 25 870 in Europe, where the medium rate of mortality is of 2,6 and the cumulated risk of 0,32%, respectively 359 died in Romania, which has a medium rate of mortality due to endometrial cancer of 1,7, with a cumulated risk of 0,2%. As expected, the medium rate of mortality is higher in the developed countries (2,3) compared to the undeveloped ones (1,5), being maximum in Europe (2,6) and minimum in South-East Asia (1,0), East-Mediterranean Region (1,2) and Africa (1,3).

According to Globocan estimates in Romania, in 2012, there were 1 539 new cases, among which, 62,24% under 65 years old. For 2015, a raise in the number of new cases with 2,92% is expected, for 2025 with 10,07% and for 2035 with 15,33%, among which under 65 years, there will be 990 new cases in 2015, 986 new cases in 2025 and 1027 new cases of endometrial cancer in 2035. For Europe, a raise with 27,2% in the number of new cases is estimated in 2035. At the world level, the new reported cases in 2012 were 319 605, among which 64,37% were women below 65 years old. The estimated number of new cases for 2015 is of 344 995, representing a growth with 7,94% compared to 2012. For 2025, the global raise in the number of new cases will be of 34,54% and for 2035 of 61,79%. The number of deaths in Romania will raise from 359 in 2012, to 370 in 2015, 399 in 2025 and 430 in 2035, the cases below 65 years old being 153 in 2015, 149 in 2025, respectively 157 in 2035, a growth in the mortality rate being noticed only over 65 years old. In 2035, 133 908 deaths due to endometrial cancer will be registered, among which 46 409 will be below 65 years old. 

In order to understand the medical and economic burden induced by endometrial cancers at the national level, these could be approached from the point of view of morbidity, mortality, medical services consumption, costs they imply for individuals, families or the health system and last but not least from the perspective of the quality of life.

**Fig. 1:**
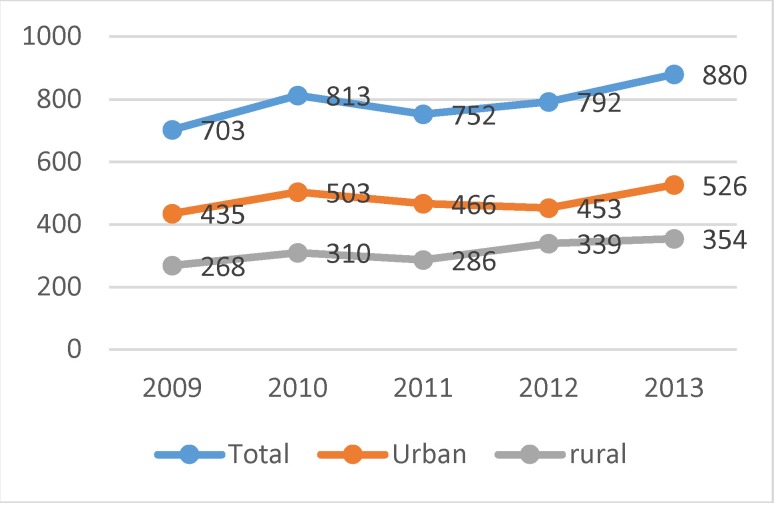
Distribution of the cases of endometrial cancer at the national level and according to the residency area, between 2009 and 2013

The analysis of the incidence based on the family physicians’ reports has been focused on the new cases of endometrial cancer, respectively code 128 from 999 classification. Overall, in the 5 years of observation, the family physicians reported 3 940 cases of endometrial cancer, among which 60.5% were from the urban area (**Fig. 1**). 

**Fig. 2:**
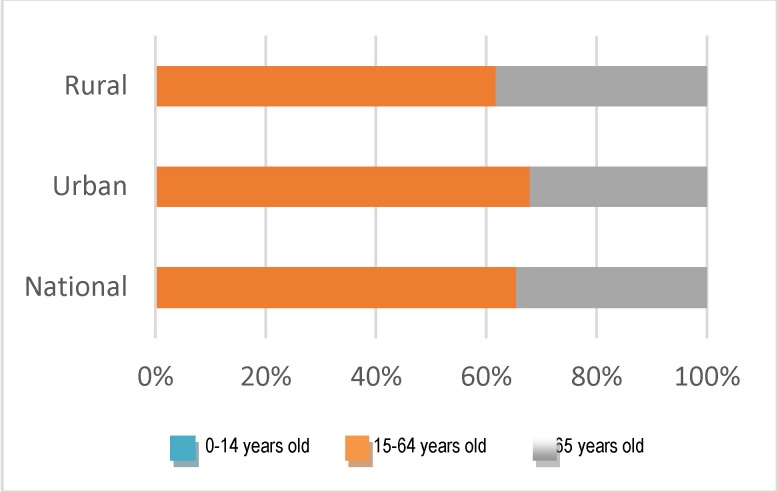
Structure of the cases of endometrial cancer according to age groups (2009-2013 cumulated)

It can be observed that approximately 800 new cases are reported annually at the national level (a little more than half compared to Globocan estimates), and, a slight tendency of the growth in the number of such cases is highlighted. The analysis of the new cases on age groups showed that in the five years which were considered, over 60% of the new cases were registered from young women (15-64 years old) (**Fig. 2**). 

**Fig. 3:**
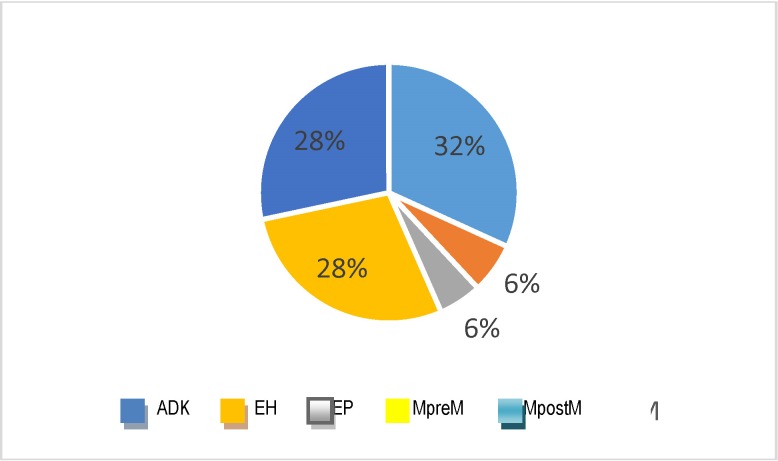
Structure of admissions due to disease causes

**Fig. 4:**
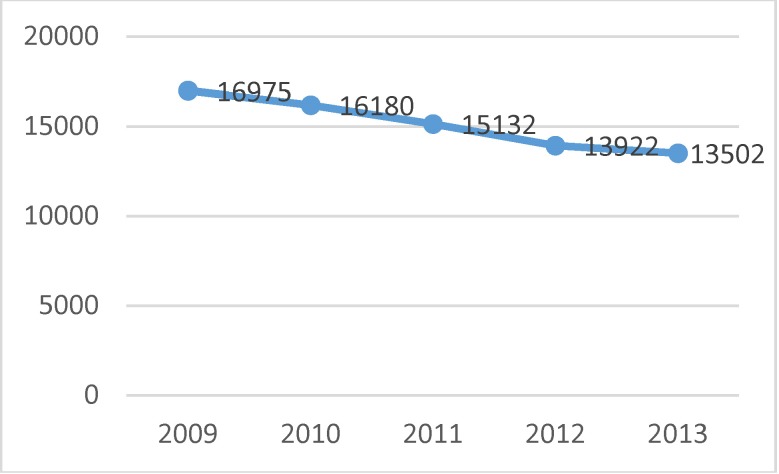
Distribution of admissions per year

During the study, sources of data such as the reports validated by the National School of Public Health and Management in Bucharest were used in order to analyze the admissions due to endometrial cancer. The diagnoses taken into consideration were the following: endometrial cancer (ADK), endometrial hyperplasia with/ without atypia (EH), endometrial polyps (EP), premenopausal metrorrhagia (MpreM) and postmenopausal metrorrhagia (MpostM). Overall, in the five years of analysis, 75 711 admissions due to this disease were registered at the national level. The structure according to the causes is presented in **Fig. 3** and the distribution per year is presented in **Fig. 4**. 

**Fig. 5:**
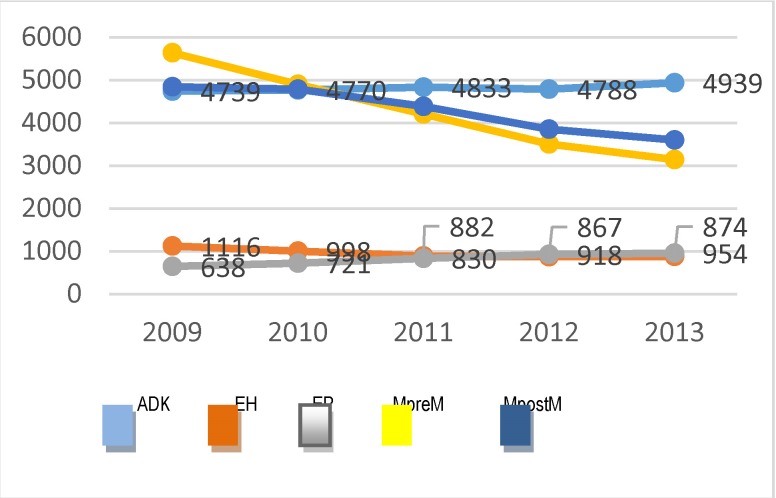
Analysis of admissions per year and main diagnosis, in Romania, between 2009 and 2013

It was observed that a third of the pathology was represented by admissions for endometrial cancer and over a half of the pre/ postclimax metrorrhagia with equal structural representations. The hyperplasia and endometrial polyps equally contributed to the formation of 12% of the investigated morbidities; taking into account the progression potential and coexistence of these lesions in the endometrial cancer, a special attention should be given to the standardized treatment and the periodic follow-up of these cases. A decrease tendency in the number of admissions can be observed from the data analysis, but this decrease has to be interpreted in the general context of the population decrease (legal population) of almost 5% during the study period and of an insufficiently known mechanical dynamics of the population. To this general context, the crisis of the health system, with the progressive diminution of the number of admissions contracted by the NHHI with the hospitals is added. Normally, the analysis of the admissions should be completed with the one of the ambulatory services offered for the five causes / symptoms analyzed, but data regarding the ambulatory services could not be identified. The analysis of the admissions per year and main diagnosis showed that, by exception from the general decrease tendency, the number of admissions due to cancer presented a slight growing tendency (**Fig. 5**). 

**Fig. 6:**
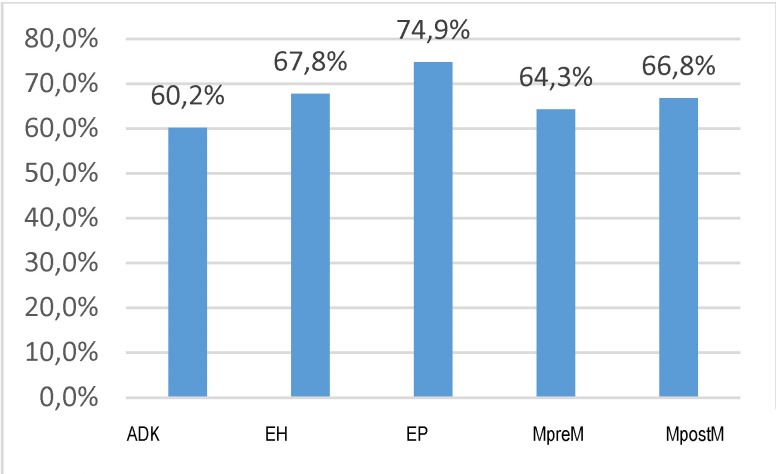
Percent of cases in urban area for each disease/ symptom

Of the total of 75 722 admissions, 48 816 were cases representing patients who live in the urban area (64.5%), which raise the problem of an inequity regarding the access to the services for women in the rural area. The percentage distribution of the cases in the urban area for each disease/ symptom is shown in **Fig. 6**. 

**Fig. 7:**
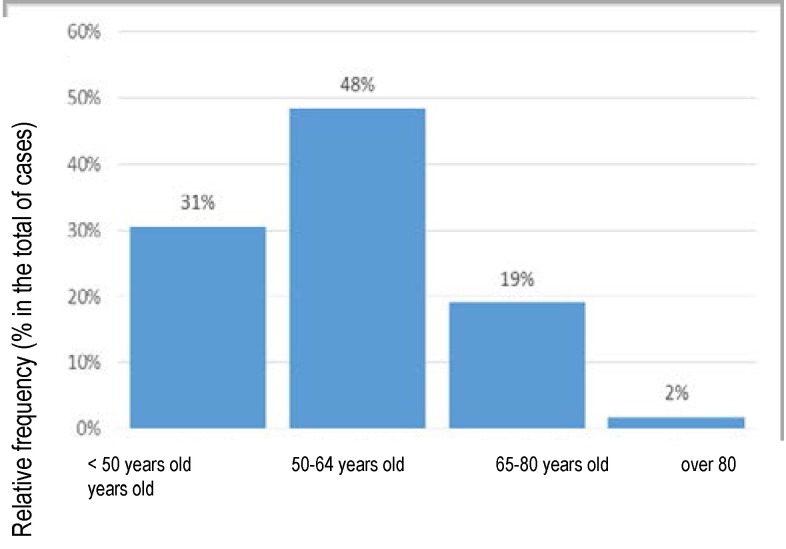
Structure of admissions according to age groups, all the causes included

The analysis of the distribution according to age groups, for the five years, shows that the social gravity of the situation, taking into account that almost a third of the admissions represent young persons (below 50 years old) and almost half represents persons aged 50-64 (**Fig. 7**).

**Fig. 8:**
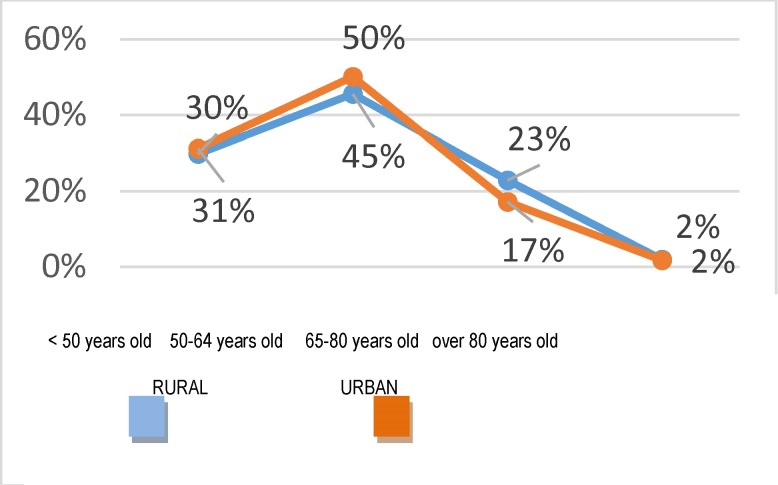
Structure of admissions according to higher age groups, all the causes included – based on residency areas

This way, the concentration of the endometrial pathology becomes obvious on the age category of 50-64 years, which should be taken into account when implementing the health programs which are to be adopted (**Fig. 8**). 

**Fig. 9:**
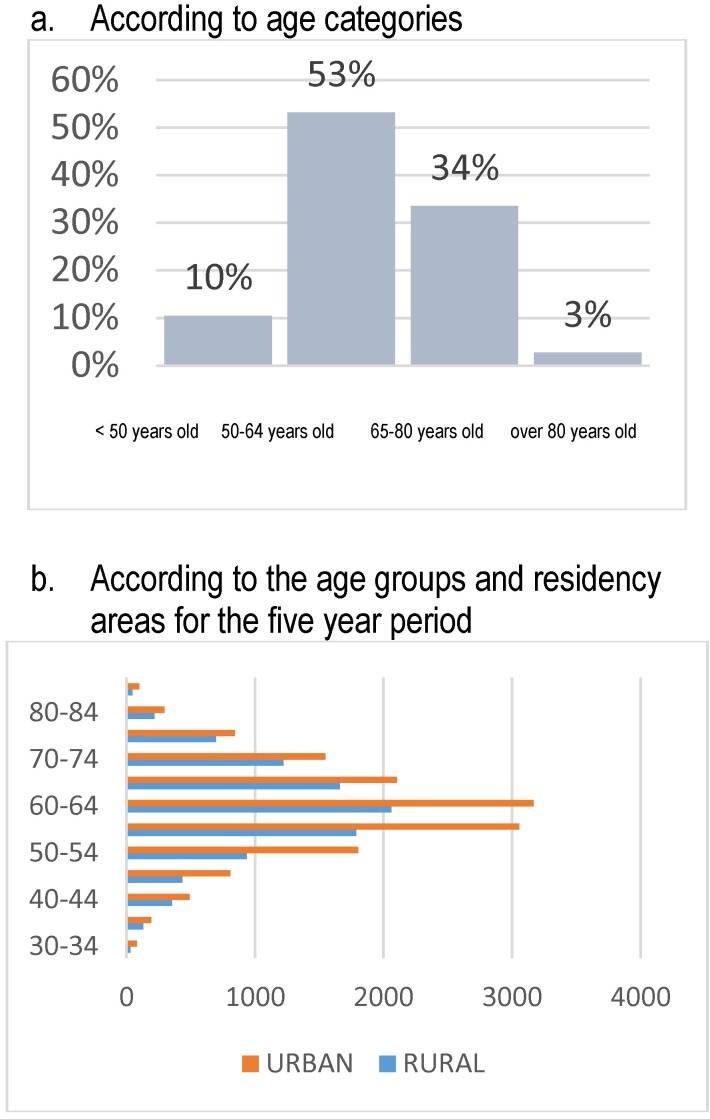
Structure of admissions – uterine adenocarcinoma

At the national level, the maximum incidence age interval for the admissions with the main diagnosis of endometrial cancer is of 60-64 years, but what should also be taken into account is the presence of this pathology with an incidence that progressively rises according to the following age groups: 30-34 years, 35-39 years, 40-44 years and 45-49 years. From the total admissions for diagnosed endometrial cancers, 10% have affected women below 50 years old and 2,78% women below 40 years old, the data in literature recording a frequency of appearance of 5% for endometrial cancer below 40 years old [**2**]. According to the recommendations of the specialty international forums, this casuistic segment is very important because the endometrial tumors present in women below 50 years old have a high risk of belonging to the category of hereditary cancers and their testing in order to establish the genetic diagnosis is compulsory due to the individual risk of these patients but also to the familial risk of developing multiple forms of cancer during lifetime (**Fig. 9**). 

Annually, the medium number of admissions with the main diagnosis of endometrial cancer is of 4 814 cases (with a light tendency of increase) and the medium number of admissions below 50 years old is of 505 cases. The absence of hereditary cancer from the national statistic data imposed the further study on the cases of colorectal cancer, whose occurrence below 50 years old raise the same degree of suspicion regarding the genetic etiology. As a result, from the family physicians’ reports it was noted, that annually, a medium number of 2 493 new cases of colorectal cancer in women (average 2009-2013) are registered, but their report was done for the following age groups: 0-14 years old, 15–64 years old, 65 years old and over 65 years old. In conclusion, the number of new cases of colorectal cancer in women below 50 years old from this source cannot be appreciated. From the study realized by the National Cancer Register regarding the new cases reported by the county oncology cabinets, on a period between 2005 and 2007, a medium value of 108 new cases of endometrial cancer per year and respectively 237 new cases of colorectal cancer in women below 50 years old per year, resulted. The cumulated figure is significant, representing the patients whose genetic testing is essential in defining an important group of high risk for familial cancer, a group of risk for which screening for endometrial cancer is universally accepted. The phenomena gain amplitude because each patient who is diagnosed with hereditary cancer requires the genetic testing of the family and the screening for endometrial cancer of the affected relatives. 

**Fig. 10:**
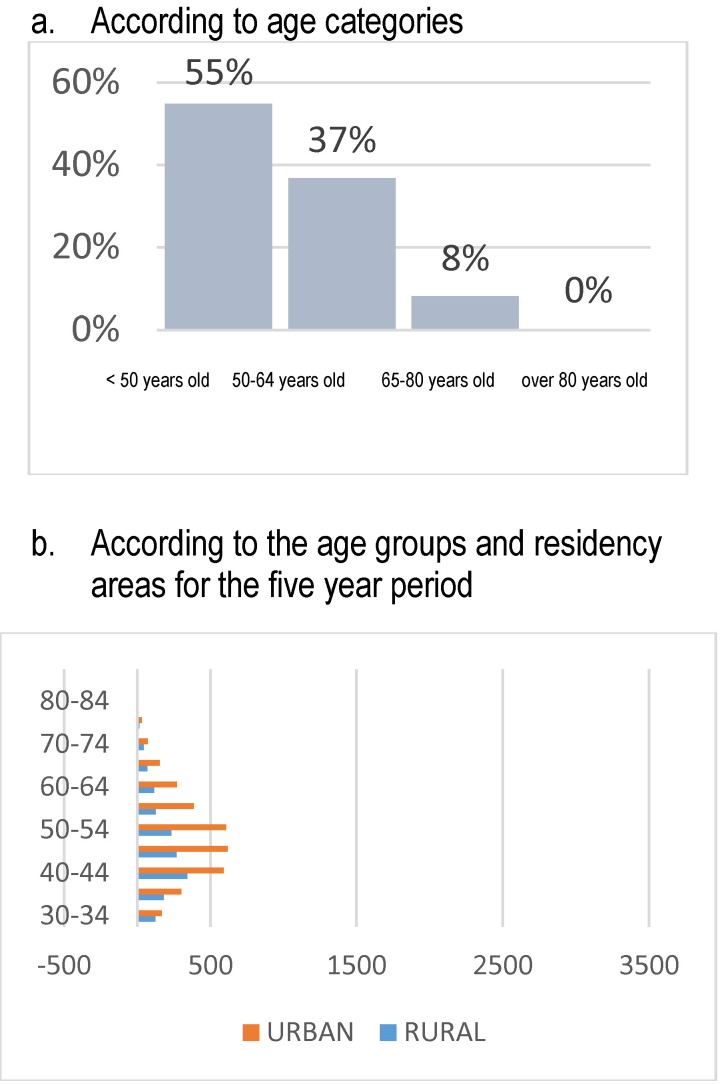
Structure of admissions – endometrial hyperplasia

Due to the fact that the reports globally included both types of endometrial hyperplasia, the peak of incidence was registered below 50 years old, 37% of the hyperplasia cases being reported in women between 50 and 64 years old (**Fig. 10**).

**Fig. 11:**
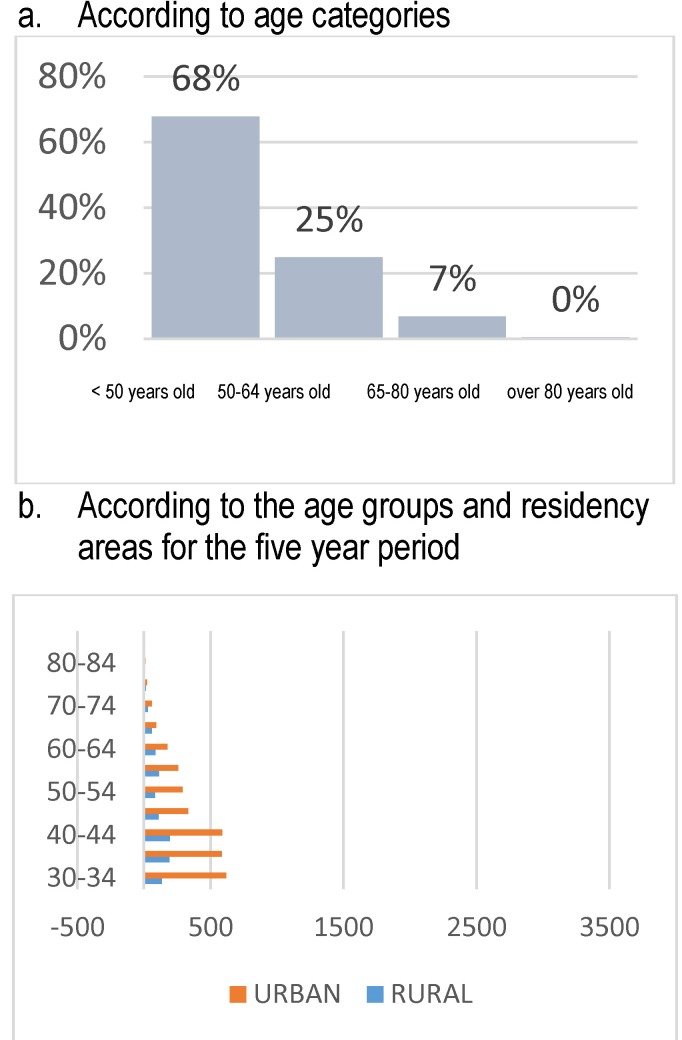
Structure of admissions – endometrial polyps

The endometrial polyps affected in 68% of cases women below 50 years old, but the most important aspect underlined by the study according to the age groups on the five-year period was that the peak incidence was registered between 30 and 34 years old. A significant decrease in the occurrence and clinical manifestation of the pathology could be noticed compared to literature data (**Fig. 11**). 

**Fig. 12:**
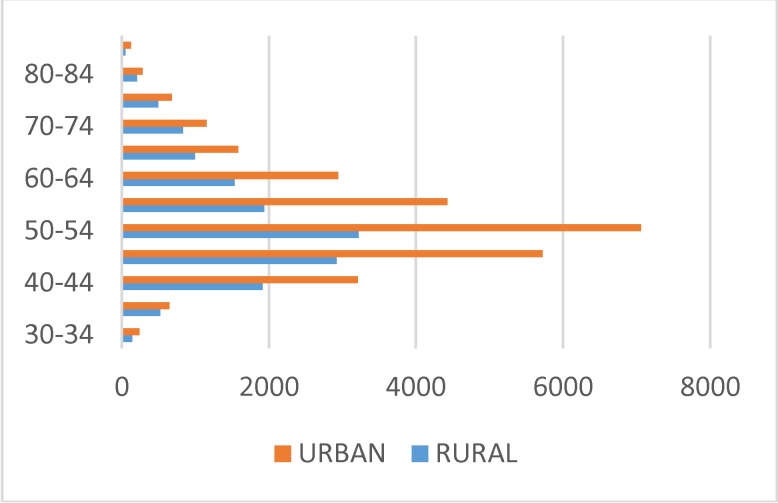
Structure of admissions – pre and post menopause metrorrhagias on age categories and residency areas on the five-year period

The analysis of the structure of admissions with the main diagnosis of metrorrhagia highlighted the concentration of this pathology on the age segment of 50 to 64 years, the peak incidence being registered in the urban area, with a significant difference as compared with the rural area in the age segment of 50 to 54 years, respectively in the period of menopause, which implied important fluctuations of the hormone levels, which were reflected at the level of the endometrium (**Fig. 12**). 

Given the fact that the first sign of endometrial cancer is the bleeding in the starting stage of the disease and, taking into account that the time passed between the occurrence of the bleeding and the treatment, seriously influence the survival rate, the reduced addressability of the women in rural area for the abnormal uterine bleeding represents a problem with serious repercussions to the prognosis of the affection in this segment of population. 

**Fig. 13:**
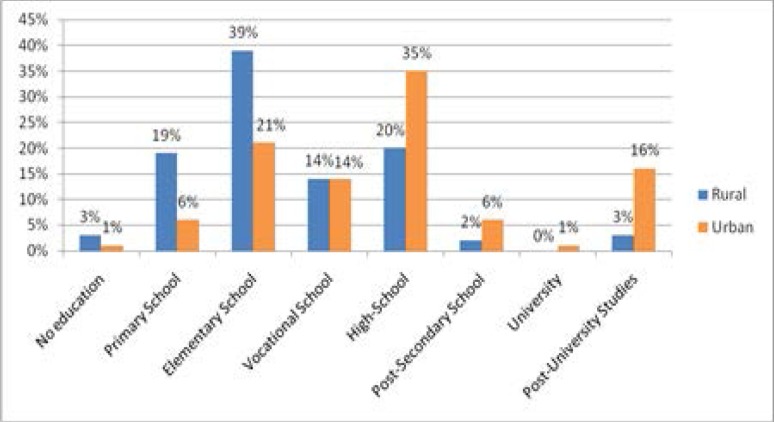
Structure of admissions according to the level of education and residency areas – all the causes included

Data regarding 75 206 cases released in the five year period were available for the analysis of the level of instruction. In a third of these cases (n=25067), the level of instruction was not mentioned. Significantly high proportions for the higher levels of education were noticed in the urban area, respectively significantly high proportions for the lower levels of education were noticed in the rural area (**Fig. 13**). 

No particular association of the admission frequency was evidenced in the cases in which the level of education was specified. 

**Fig. 14:**
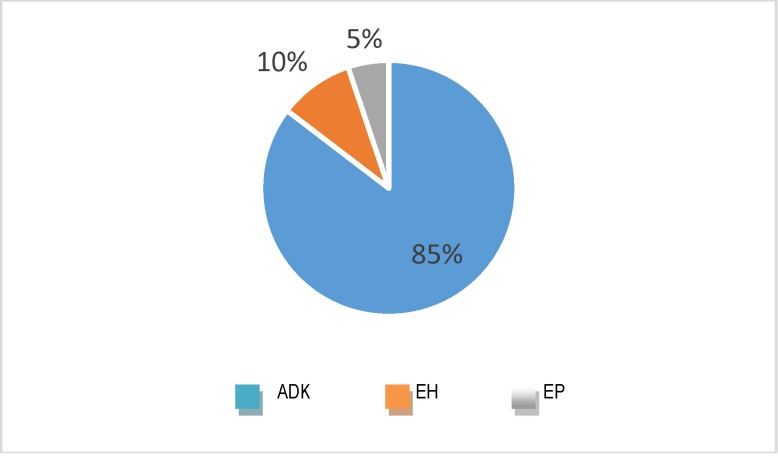
Diseases in which risk factors were noted – global level

The followed risk factors were diabetes, obesity and hypertension and were noted in the case of 16 674 admissions, among which, 85% were endometrial cancers (**Fig. 14**).

**Fig. 15:**
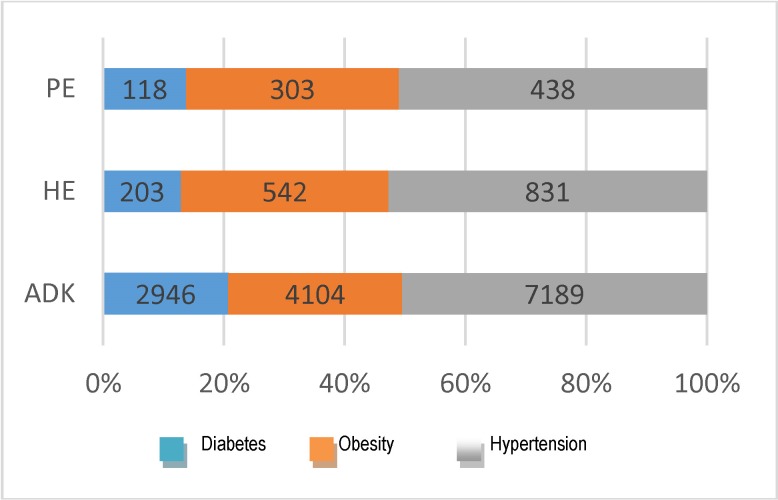
Percentage of risk factors in each disease

The most independent pathology of the analyzed risk factors was the endometrial polyps, while the hypertension-obesity-diabetes triad represented the specific etiopathogenic aspect of type I endometrial cancer, which is dependent on estrogen and represents 80% of the total types of endometrial cancer [**3**]. Hypertension was noted to be the most frequently met (51% of the cases, n=8458). By analyzing the percentage of the risk factors according to each endometrial pathologic entity, it was noticed that obesity and hypertension were found in relatively constant percentages as compared to all the 3 endometrial affections, but the percentage of diabetes was considerably higher in the endometrial cancer cases, the excess adipose tissue being responsible for the connection of hyperestrogenism with the glucidic metabolic imbalance from an etiopathogenic point of view (**Fig. 15**). 

The analysis of the diseases’ exposure rates showed that 59% of the endometrial cancer cases presented at least one of the analyzed risk factors. The difference between the malignant and the benign pathology of the endometrium was visibly reflected in the global and individual association of the studied comorbidities [**4**].

**Fig. 16:**
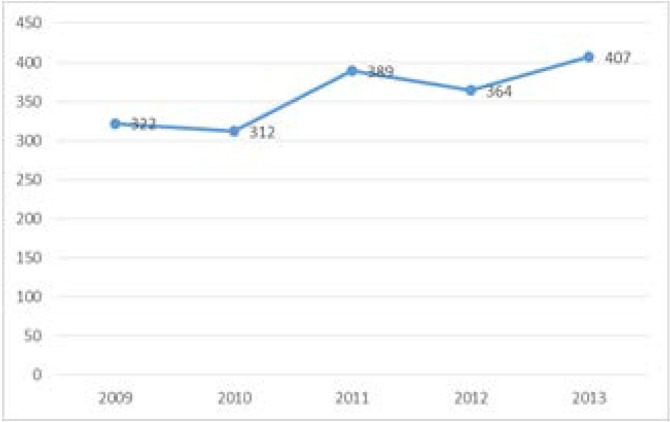
Evolution of deaths due to endometrial cancer in Romania, 2009-2013

During the analyzed period, 1794 deaths due to endometrial cancer were registered, with an average of 359 deaths / year and a net trend of increase (**Fig. 16**). 

**Fig. 17:**
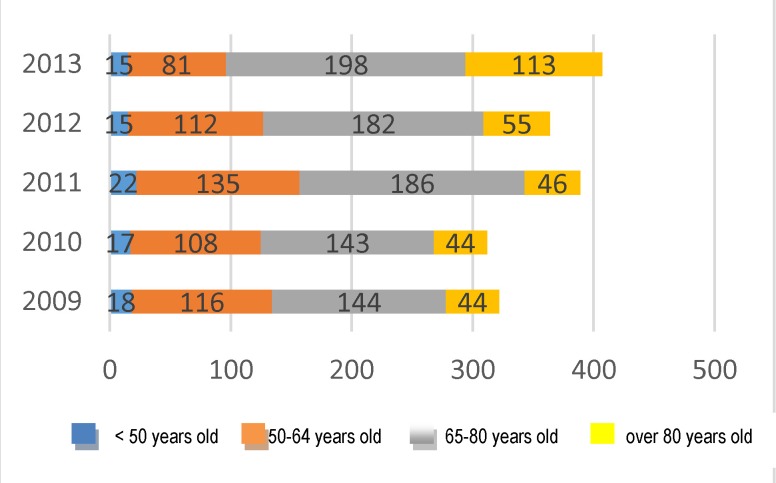
Structure of deaths according to age groups

Moreover, it was noticed that, for each year, most of the deaths were registered in the following age groups: 65-79 years (n=853 during the five-year period) and 50-64 years (n=552 during the whole period) (**Fig. 17**). 

During the five-year period, 302 deaths were registered in women over 80 years old, and 87 deaths in women below 50 years old. Although they seemed few, the latter raised an important issue due to the age of occurrence, the mortality rate below 50 years old representing the corroboration of a precocious age of neoplastic affection with an advanced stage of the disease’s diagnosis, the presence of some aggressive histopathologic forms and the existence of other adverse prognostic factors of the disease’s evolution. 

The analyzed data offered a partial picture regarding the impact of precancerous pathology and endometrial cancer on morbidity, mortality and the use of medical services. There are no studies that can highlight the costs implied by this pathology for persons, families or the health system, and finally yet importantly, there are no analyses regarding the survival rates in Romania or the evaluation of the quality of life. 

Although fragmentary, from the analyses presented, it could be noticed that despite the fact that it is considered a rare disease, endometrial cancer and precancerous pathology have serious implications in women’s health state, and, specifically for Romania, starting before the age of 50. As perspective recommendations, it can be affirmed on one side that it is important to improve the informational system to include all the aspects of this disease and, on the other side it is compulsory to assure the functionality of the National Cancer Registry.
